# Dp71 Expression in Human Glioblastoma

**DOI:** 10.3390/ijms20215429

**Published:** 2019-10-31

**Authors:** Simona Ruggieri, Michelina De Giorgis, Tiziana Annese, Roberto Tamma, Angelo Notarangelo, Andrea Marzullo, Rebecca Senetta, Paola Cassoni, Michela Notarangelo, Domenico Ribatti, Beatrice Nico

**Affiliations:** 1 Department of Basic Medical Sciences, Neurosciences and Sensory Organs, University of Bari Medical School, 70124 Bari, Italy; simona.ruggieri@uniba.it (S.R.); michelina.degiorgis@uniba.it (M.D.G.); tiziana.annese@uniba.it (T.A.); roberto.tamma@uniba.it (R.T.); 2 Medical Genetic Unit, IRCCS Casa Sollievo della Sofferenza, San Giovanni Rotondo, 71013 Foggia, Italy; a.notarangelo@operapadrepio.it; 3 Department of Emergency and Transplantation, Pathology Unit, University of Bari Medical School, 70124 Bari, Italy; andrea.marzullo@uniba.it; 4 Pathology Unit, Department of Medical Sciences, University of Turin, University of Turin Medical School, 10124 Turin, Italy; rebecca.senetta@unito.it (R.S.); paola.cassoni@unito.it (P.C.); 5 Centre for Integrative Biology, CIBIO, University of Trento, 38123 Trento, Italy; michela.notarangelo@unitn.it

**Keywords:** Dp71, glioblastoma, lamin B, tumor progression

## Abstract

Background: Dp71 is the most abundant dystrophin (*DMD*) gene product in the nervous system. Mutation in the Dp71 coding region is associated with cognitive disturbances in Duchenne muscular dystrophy (DMD) patients, but the function of dystrophin Dp71 in tumor progression remains to be established. This study investigated Dp71 expression in glioblastoma, the most common and aggressive primary tumor of the central nervous system (CNS). Methods: Dp71 expression was analyzed by immunofluorescence, immunohistochemistry, RT-PCR, and immunoblotting in glioblastoma cell lines and cells isolated from human glioblastoma multiforme (GBM) bioptic specimens. Results: Dp71 isoform was expressed in normal human astrocytes (NHA) cell lines and decreased in glioblastoma cell lines and cells isolated from human glioblastoma multiforme bioptic specimens. Moreover, Dp71 was localized in the nucleus in normal cells, while it was localized into the cytoplasm of glioblastoma cells organized in clusters. We have shown, by double labeling, that Dp71 colocalizes with lamin B in normal astrocytes cells, confirming the roles of Dp71 and lamin B in maintaining nuclear architecture. Finally, we demonstrated that decreased Dp71 protein in cells isolated from human bioptic specimens was inversely correlated with the Ki-67 tumor proliferative index. Conclusion: A decreased Dp71 expression is associated with cancer proliferation and poor prognosis in glioblastoma.

## 1. Introduction

Dp71 is the most abundant product of the dystrophin (*DMD*) gene in the adult brain [1]. The *DMD* gene consists of 79 exons and seven alternative promoters used to express tissue-specific dystrophin isoforms. Dp71 is a 71 kDa protein transcribed from a promoter located in intron 62 [2]. It has been reported that mutations in the Dp71 coding region are associated with cognitive impairment in Duchenne muscular dystrophy (DMD) patients [3,4,5]. Furthermore, the expression of Dp71 is involved in the development of the nervous system [6], and current Dp71 research identifies its involvement in ion and water homeostasis, cell signaling, cell adhesion, and nuclear architecture [2,7,8,9,10]. Little data are available concerning the role of Dp71 in tumor progression. In gastric cancer, Dp71–lamin complexes were found to have tumor suppressive effects [11]. In addition, knockdown of Dp71 was found to reduce malignancy of a lung adenocarcinoma cell line [12]. Recently, Rani et al. [13] have identified six splice variants of Dp71 in glioblastoma cells, with Dp71b being the most abundant.

In this study, we analyzed Dp71 and lamin B expression in glioblastoma multiforme (GBM), the most common and aggressive primary tumor of the central nervous system (CNS), which is characterized by rapid proliferation, metastatic capacity-enhanced angiogenesis, and resistance to treatment [14,15,16].

We demonstrated using immunofluorescence, immunohistochemistry, RT-PCR, and immunoblotting that Dp71 isoform is expressed in normal human astrocytes (NHA) cell lines and decreased in glioblastoma cell lines and in cells isolated from human GBM bioptic specimens. Moreover, Dp71 was localized in the nucleus of normal cells, while it was localized in the cytoplasm of glioblastoma cells and organized in clusters. We have shown, by double labeling, that Dp71 colocalizes with lamin B in normal astrocytes cells, confirming the roles of Dp71 and lamin B in maintaining nuclear architecture. Finally, we demonstrated that decreased Dp71 protein in cells isolated from human bioptic specimens inversely correlated with the Ki-67 tumor proliferative index.

## 2. Results

### 2.1. Subcellular Reorganization of Dp71 Protein in Glioblastoma and Meningioma Cells and Interaction between Dp71 and Lamin B in NHA Cells

**Figure 1:**
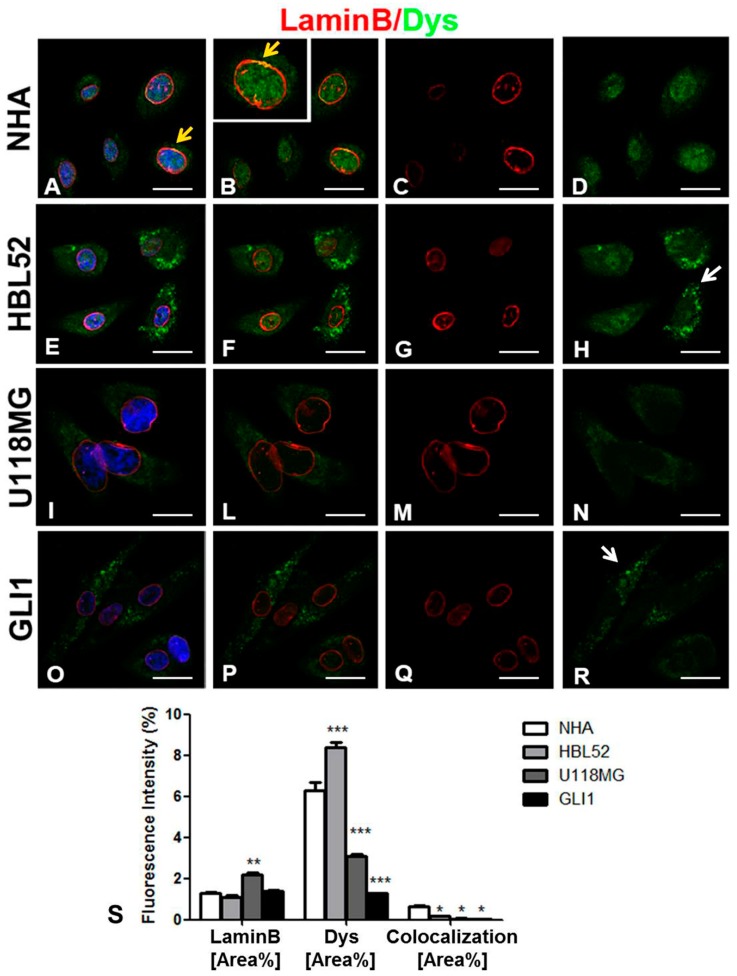
Lamin B (red) and Dp71 dystrophin isoform (Dys) (green) confocal dual immunofluorescence reaction in tumor (**E**–**I**,**L**–**R**) and normal human astrocytes (NHA) (**A**–**D**) cells. Dys fluorescent signal decreases and localizes in the cytoplasm of U118MG (**I**,**L**,**N**) and the glioblastoma primary cell line (GLI1) (**O**,**P**,**R**) tumor cells compared with strong and diffused nuclear signal in the NHA control cells (**A**,**B**,**D**). An orange fluorescence signal corresponding to colocalization of lamin B and Dys is present in NHA cells (**A**,**B** yellow arrows). HBL52 meningioma cells (**E**–**H**) show a strong cytoplasmic and clustered Dys signal (**E**,**F**,**H**, white arrow) compared to the control cells (**A**,**B**,**D**). Lamin B red fluorescence increases in U118MG (**L**,**M**) tumor cells compared to the NHA control cells (**B**,**C**). Morphometric analysis (**S**) shows a significant reduction of Dys fluorescence intensity in U118MG and GLI1 tumor cells, a significant increase of Dys expression in HBL52 meningioma cells, and a significant increase of lamin B expression in U118MG cells compared to NHA cells. The fluorescence intensity of colocalization decreases in tumor cells. (* *p* < 0.05, ** *p* < 0.01, *** *p* < 0.001—all groups vs. NHA group) Scale Bar: A–R 10 µm.

We analyzed the expression of Dp71 protein in glioblastoma cells, including the U118MG cell line and in glioblastoma cells obtained from three patients with GBM. We also analyzed Dp71 expression in the HBL52 cell line (derived from transitional meningioma grade I) to evaluate the correlation of Dp71 expression with the malignancy grade. All of the analyzed cell groups were compared with the NHA cell line. A dual confocal immunofluorescence reaction of Dp71 and lamin B in glioma and NHA cells was performed (Figure 1A–R). The results show that Dp71 protein was present in the nucleus of NHA cells (Figure 1A,B,D) and colocalizes with lamin B in a nuclear envelope (Figure 1A,B yellow arrow). The Dp71 protein pattern was different in glioblastoma and meningioma cells. The primary glioblastoma cells (GLI1) and HBL52 cells show a Dp71 cytoplasmic expression with a formation of clusters (Figure 1E,F,H,O,P,R black arrow) and no colocalization signal between Dp71 and lamin B proteins was detected (Figure 1E–I,L–R). Moreover, Dp71 expression was reduced in U118MG and in GLI1 (Figure 1I,L–R), while lamin B expression was increased in the U118MG cell line in association with the increased size of the tumor cell nuclei (Figure 1I,L,M) Morphometric analysis confirmed a meaningful reduction of Dp71 fluorescence intensity in U118MG and GLI1 cells, while Dp71 increased in HBL52 cells compared to control NHA cells (Figure 1S). Lamin B was overexpressed only in the U118MG cell line and lamin B/Dp71 dystrophin isoform (Dys) did not colocalize (Figure 1S).

### 2.2. Decreased Dp71 and Increased Lamin B mRNA and Protein Expressions in U118MG Cell Line and in Glioblastoma Cells Derived from GBM Patients

**Figure 2:**
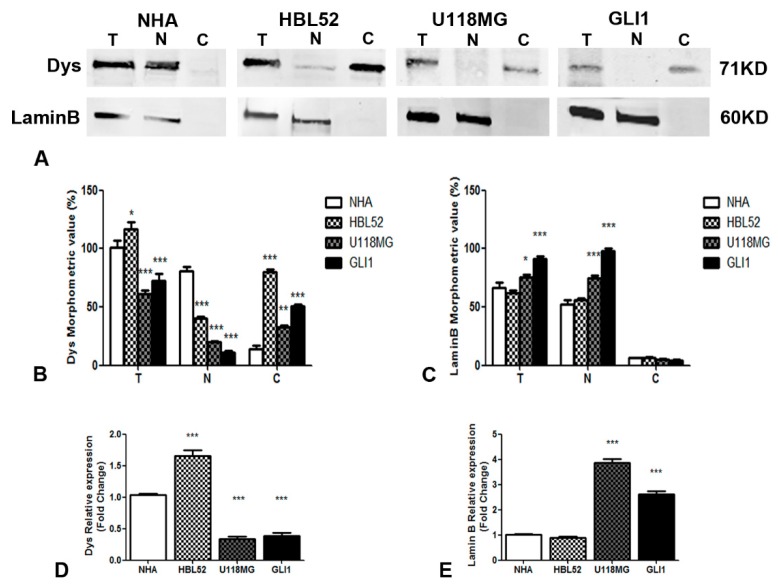
Lamin B and Dys protein (**A**–**C**) and mRNA expression (**D**,**E**) in tumor and NHA cells. Western blotting analysis shows Dys cytoplasmic (**C**) expression in HBL52, U118MG, and GLI1, tumor cells compared with Dys nuclear (**N**) expression in NHA cells (**A**). The lamin B expression is only nuclear for the presence of equal total (**T**) and nuclear (**N**) bands (**A**). The quantification of Dys bands (**B**) shows a significantly lower level of Dys proteins in total (**T**), nuclear (**N**), and cytoplasmic (**C**), protein fractions of U118MG and GLI1 cells compared to NHA cells, while the total (**T**) and cytoplasmic (**C**) protein fraction increases in HBL52 cells compared to NHA cells (**B**). The quantification of lamin B bands (**B**) reveals a significantly higher level of lamin B proteins in total (T) and nuclear (N) protein fractions of U118MG and GLI1 cells compared to NHA cells (**C**). The bands are representative of three independent experiments in triplicate for each protein, and β-actin was used as a housekeeping gene. The data are expressed as proteins/β-actin ratio ± SEM. The Dys mRNA expression analysis by real-time PCR (D) shows a significant increase of Dys mRNA levels in HBL52 cells and a decrease in U118MG and GLI1 cells as compared with the control NHA cells (D). The lamin B mRNA expression analysis by real-time PCR (E) shows a significant increase of lamin B mRNA levels in U118MG and GLI1 cells as compared with the control NHA cells (E). (* *p* < 0.05 all groups vs. NHA group; ** *p* < 0.01 all groups vs. NHA group; *** *p* < 0.001 all groups vs. NHA group).

Western blot analysis was performed using total, cytoplasmic, and nuclear extract proteins to determine Dp71 and lamin B protein levels and to quantify the difference in subcellular Dp71 expression in NHA, HBL52, and U118MG cell lines, and in glioblastoma primary cells (Figure 2A–C). The Dp71 total expression level was significantly lower in U118MG and GLI1, and significantly higher in HBL52 compared with the NHA control cell line. Moreover, Dp71 protein levels were decreased in the nucleus and increased in the cytoplasm of HBL52, U118MG, and GLI1, compared to the control (Figure 2A,B). Otherwise, nuclear lamin B expression was increased in glioma cells (Figure 2A,C). Immunofluorescence and Western blot demonstrated that nucleocytoplasmic shuttling of Dp71 protein occurs in oncogenic conditions.

Real-time quantitative PCR was performed to determine the Dp71 and lamin B mRNA levels in glioblastoma cells. Dp71 expression was lower in U118MG and in GLI1 cells, and increased in HBL52 cells compared to the controls (Figure 2D). The increased lamin B mRNA levels were only detected in U118MG and GLI1 cells (Figure 2E).

### 2.3. Decreased Dp71 Expression in Glioblastoma Bioptic Specimens and Correlation with the Ki-67 Tumor Proliferative Index

**Figure 3:**
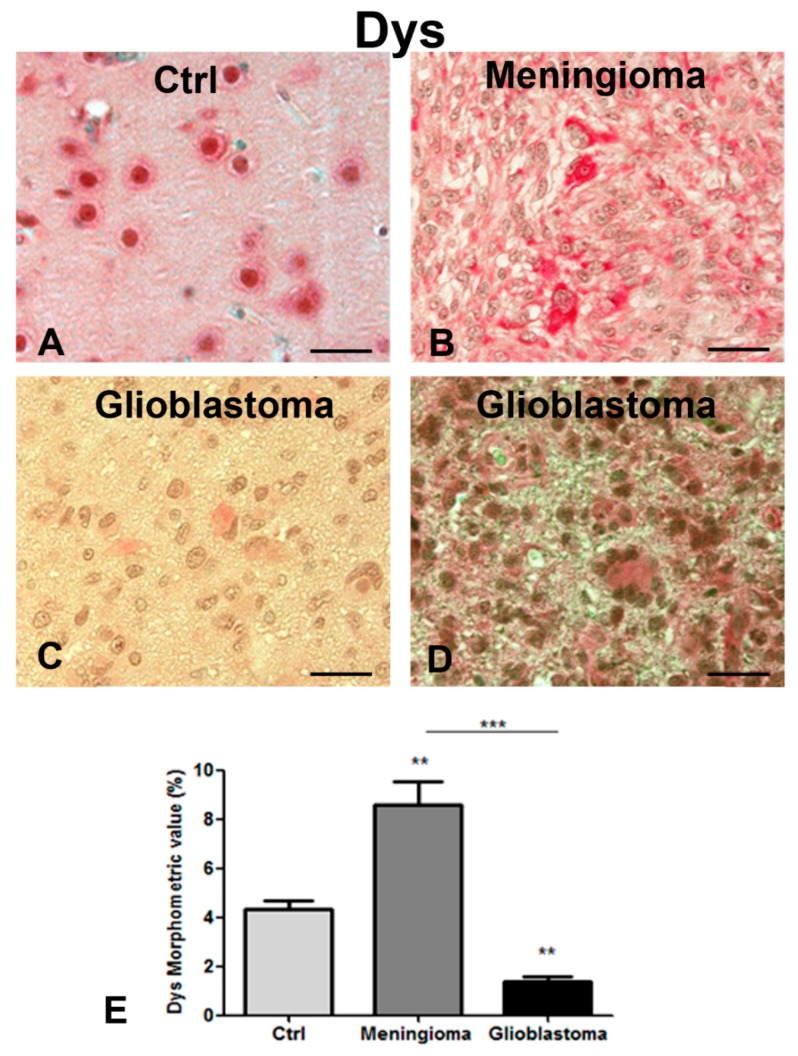
Dys immunohistochemistry analysis in the tumor (**B**–**D**) and control (**A**) bioptic specimens. Strong nuclear staining is detectable in the control section (**A**) while a diffuse and intense cytoplasmic signal is present in meningioma cells (**B**). Glioblastoma cells (**C**,**D**) show a light Dys cytoplasmic expression with no nuclear staining. Morphometric analysis (**E**) shows a significant decrease of Dys protein in glioblastoma cells compared to the meningioma and control ones, while the meningioma section shows a significant increase of Dys protein level compared to the control section. (** *p* < 0.01 vs. control; *** *p* < 0.001 vs. meningioma). Scale bar **A**,**B**,**C**,**D**: 20 μm.

Immunohistochemistry analyses were carried out on meningioma and glioblastoma specimens (Figure 3A–E). Dp71 was localized prevalently in the nucleus of the control sections (Figure 3A). In meningioma, a strong cytoplasmic Dp71 expression was observed (Figure 3B) while, in glioblastoma, cytoplasmic localization was associated with a significant reduction of Dp71 expression (Figure 3C,D) compared with meningioma (Figure 3B) and the controls (Figure 3A). Morphometric analysis showed an overexpression of Dp71 protein in meningioma and a reduction in glioblastoma (Figure 3E). Furthermore, we correlated Dp71 expression with the Ki-67 tumor proliferative index in glioblastoma specimens. As shown in Table 1, a higher expression was found in patients with a higher Ki-67 index, suggesting that Dp71 expression is associated with cell proliferation.

## 3. Discussion

In this study, we have demonstrated, for the first time, a significant reduction of the Dp71 mRNA and protein in glioblastoma cell lines and in cells isolated from human GBM bioptic specimens compared with the astrocytic control cell line. There was subcellular reorganization with a cytoplasmic and clustered expression pattern for Dp71 protein in glioblastoma cells and in meningioma cell lines as compared with nuclear and diffuse signal localization in the control cell line, where Dp71 colocalized with lamin B. These results confirm that, in normal conditions, Dp71 participates in nuclear architecture, serving as scaffolding for nuclear processes [17].

In normal conditions, Dp71—linked with dystrophin-associated proteins (DAPs) as aquaporin-4 (AQP4), alpha/beta-dystroglycan complex, and agrin—is highly expressed by perivascular astrocytic endfeet, where it controls the integrity and function of the blood–brain barrier [18].

An altered expression pattern of Dp71- and DAP-associated proteins was found in GBM [19]. AQP4 proteins are involved in cell migration, angiogenesis, and edema formation in human brain tumors [20,21]. The α-dystroglycan (α-DG) protein may be involved in the progression of primary brain tumors [22].

There are two Dp71 isoforms that are derived from alternative splicing of exon 78 that show differential subcellular localization [23]. The Dp71d isoform, which includes the region encoded by exon 78, exhibits a predominant nuclear localization, while the Dp71f isoform, harboring an alternative 31-amino acid C-terminal domain by the removal of exon 78, is exclusively cytoplasmic [23,24]. The finding of the same isoform in the cytoplasm of tumor cells indicates that Dp71 can shuttle between the nucleus and cytoplasm as modulated by specific tumor stimuli [25]. The reduction in Dp71 expression and subcellular reorganization with cluster formation in glioblastoma cells suggest that Dp71 proteins have different nuclear and cytoplasmic functions.

Lamin B is an important member of the lamin protein family and is involved in crucial nuclear processes, including nuclear morphology, heterochromatin organization, cell division, and senescence [26,27,28,29]. The role of lamin B in cancer development and progression is unclear and controversial. In this study, we demonstrated that lamin B was significantly overexpressed in glioblastoma cell lines compared to controls.

The role of lamin B in cancer development and progression is controversial [30,31,32]. The expression of lamin B is reduced in lung cancer, colon cancer, and gastric cancer [11,33,34], whereas its expression is increased in prostate cancer, hepatocellular carcinoma, and pancreatic cancer [35,36,37,38]. Our data have shown that lamin B expression was significantly increased on the nuclear envelope of the glioblastoma cell line and patient glioma cells as compared with the controls. The change in lamin B expression was not detected in the meningioma cell line, suggesting that its expression correlates with an increase in glioma malignancy grade.

Finally, we demonstrated that decreased Dp71 protein in cells isolated from human bioptic specimens were inversely correlated with the Ki-67 tumor proliferative index. The patients with higher Ki-67 exhibited lower Dp71 expression.

## 4. Materials and Methods

### 4.1. Tissue Samples

Primary human brain bioptic specimens (30 grade IV glioblastomas, 10 meningiomas, and 5 healthy tissues) were retrospectively selected from the archive of the Section of Pathology of the University of Torino, Medical School, and the Section of Pathology of the Department of Emergency Surgery and Organ Transplantation of the University of Bari, Medical School (Table 1). All procedures followed were in accordance with the ethical standards of the responsible committee on human experimentation (institutional, n.555, 5 June 2018) and with the Helsinki Declaration of 1964. All primary tumors were obtained from patients who had undergone no prior treatment (radiotherapy or chemotherapy). The tumor types and stages were determined according to the World Health Organization classification [39].

### 4.2. Cell Culture

The U118MG cell line was obtained from ATCC (American Tissue Culture Collection, Manassan,VA, USA), HBL52 cell line from CLS (Cell Lines Service, GmbH, Eppelheim, Geramany), and NHA (Lonza Group Ltd, Basel, Switzerland cat ≠ CC-2565). The U118MG cells were cultured in DMEM plus 4.5 g/L D-glucose (GIBCO by Thermo scientific^TM^, Waltham, MA, USA), while the HBL52 and NHA cells were cultured in DMEM/F-12 (1:1) (GIBCO by Thermo scientific^TM^, Waltham, MA, USA) supplemented with 10% fetal bovine serum (FBS; GIBCO, by Thermo scientific^TM^, Waltham, MA, USA), 100 U/mL penicillin, and 100 µg/mL streptomycin, and maintained at 37 °C in a 5% CO_2_ humidified atmosphere.

### 4.3. Establishment of Primary Culture

We obtained the primary culture from three patients with grade IV GBM. After surgical removal, upon the approval of the institutional Ethics Committee, tumor specimens were placed in DMEM-F12 medium without serum, repeatedly washed with phosphate-buffered saline (PBS, Invitrogen, Carlsbad, CA, USA), and then placed in a 30 mm Petri dish. The specimens were cut in 1–2 mm^3^ fragments and transferred in a poly-D-lysine treated flask with a small amount of Dulbecco’s modified Eagle’s medium/F12 medium (D-MEM/F12, GIBCO by Thermo scientific^TM^, Waltham, MA, USA) (1:1, *v/v*), supplemented with 10% fetal bovine serum (FBS, GIBCO by Thermo scientific^TM^, Waltham, MA, USA), 100 U/mL penicillin, and 100 μg/mL streptomycin (PenStrep, GIBCO by Thermo scientific^TM^, Waltham, MA, USA), in order to allow the fragments to adhere to the surface of the flask. The primary culture was incubated at 37 °C in a 5% CO_2_ humidified atmosphere, and 5 mL of complete medium was added 24 h later. After one week, the primary culture was washed with PBS to remove non-adherent fragments. Then, fresh medium, pre-warmed at 37 °C, was added. These procedures were repeated every 3 days until the primary culture reached local confluence. Then, the cells were treated with 0.05% trypsin (GIBCO by Thermo scientific^TM^, Waltham, MA, USA) and 0.02% EDTA (GIBCO by Thermo scientific^TM^, Waltham, MA, USA), washed with PBS, and transferred into a T-75 flask without biocoat containing the complete medium DMEM/F12. 

### 4.4. Real-Time PCR

RNA was extracted from cells using an RNeasy Mini Kit (Qiagen, Germantown, MD, USA), which was then used to synthesize the first strand c-DNA with the IScript cDNA Synthesis kit (Bio-Rad Laboratories, Hercules, CA, USA), according to the manufacturer’s instructions. cDNA was amplified with the iTaq SYBR Green Supermix (Bio-Rad Laboratories, Hercules, CA, USA). PCR amplification and real-time detection were performed with the Chromo4 Real-Time PCR Detection System (Bio-Rad Laboratories, Hercules, CA, USA). The expression of mRNA for Dp71 and lamin B was evaluated by real-time PCR and the samples were normalized to *cyclophilin A* as a housekeeping gene. The primer sequences are reported in Table 2. The analysis was performed with Bio-Rad CFX manager 3.1 software.

### 4.5. Western Blotting

To obtain total protein, extract tumors and control cells were homogenized in a lysis buffer (cod. 89900, Thermo Scientific Rockford, IL, USA) and protease inhibitors were incubated three times at −20 °C for 10 min. The cells were centrifuged at 14,000 rpm for 20 min and the supernatant was collected. Moreover, harvested tumor and control cells were fractionated into cytoplasmic and nuclear fractions using a nuclear and cytoplasmic extraction kit (NE-PER^TM^ Nuclear and Cytoplasmic Extraction Reagent, Thermo Fisher Scientific INC., Rockford, IL, USA). The protein concentration was determined using the detergent-compatible Bio-Rad DC protein assay (Bio-Rad Laboratories, Hercules, CA, USA). For immunoblotting, 30 mg per lane of protein extract was solubilized in Laemmli buffer, boiled at 90 °C for 5 min, and resolved on Mini-Protean TGX Precast gels (cod. 456-1104, Bio-Rad Laboratories, Hercules, CA, USA). Thereafter, the proteins were electrotransferred to a nitrocellulose membrane (Trans Blot Turbo Transfer Pack cod. 170-4158, Bio-Rad Laboratories). The blots were blocked with a PBS blocking buffer containing 5% nonfat dry milk for 1 h and incubated overnight at 4 °C with the following primary antibodies: Dys, lamin B, and β-actin (Table 3). After the primary antibody treatment, the membranes were washed 4 × 5 min each at room temperature in PBS with 0.1% Tween-20 before the addition of secondary antibodies. The PBS and 0.1% Tween-20-diluted secondary antibodies (anti-mouse and -rabbit) were IRDye labeled (800 CW) (LI-COR Biosciences, Lincoln, NE, USA). For immunoblotting analysis, the LI-COR Odyssey infrared imaging system was used (LI-COR Biosciences, Lincoln, NE, USA). The Western blot images were analyzed by imaging densitometry using ImageJ and quantified through normalization with actin and expressed as optical density mm^2^. The images are representative of three independent experiments performed in triplicate.

### 4.6. Dual Immunofluorescence Confocal Laser Scanning Microscopy

The tumor and control cells were fixed in 4% paraformaldehyde (PFA), rinsed with phosphate-buffered saline (PBS, cod. P4417, Sigma-Aldrich, St. Louis, MO, USA) and then exposed to primary antibodies diluted in PBS with 0.2% BSA (Table 3). Then, the cells were incubated for 45 min with the corresponding secondary antibodies at 37 °C (Table 3), and, following washing, they were incubated for 20 min with 0.01% TO-PRO-3 (Invitrogen, Carlsbad, CA, USA) for nuclear staining, and mounted in Vectashield (Vector Laboratories, Burlingame, CA, USA). The cells were examined under a Leica TCS SP2 (Leica, Wetzlar, Germany) confocal laser scanning microscope using 40× and 63× objective lenses with either 1× or 2× zoom factors. A sequential scan procedure was applied during image acquisition of the two fluorophores. Confocal images were taken at 200 nm intervals through the z axis of the section. The images from individual optical planes and multiple serial optical sections were analyzed, digitally recorded, and stored as TIFF files using Adobe Photoshop software (Adobe Systems Inc. San Jose, CA, USA).

### 4.7. Dys Immunohistochemistry

Four-micrometer-thick, 4% PFA-fixed and paraffin-embedded histological sections collected on poly-L-lysine-coated slides (cod. J2800AMNZ, Gerhard Menzel, Germany) were deparaffinized and rehydrated in a xylene-graded alcohol scale and then rinsed for 10 min in PBS. The sections were heated in a solution of sodium citrate pH 6.0 (cod. S1699, Dako Corporation, Milano, Italy) once the temperature had reached 98 °C in a water bath for 20 min and, after washing in TBS/Triton, were kept for 10 min in a Dual Endogenous Enzyme Block buffer (Code S2003, Dako Corporation, Milano, Italy). Afterwards, the sections were exposed to Dys primary unconjugated antibodies (see Table 3) diluted in Dako antibody diluent (cod. S3022, Dako Corporation, Milano, Italy) for 30 min at 4 °C. Immunodetection was performed with Dako REAL™ Detection System, Alkaline Phosphatase/RED (Cod.K5005, Dako Corporation, Milano, Italy). Thereafter, the sections were counterstained with Mayer’s hematoxylin and mounted in synthetic medium. The specific preimmune serum (Dako Corporation, Milano, Italy, in place of the primary antibodies, served as a negative control.

### 4.8. Morphometric Analysis

Morphometric analysis was performed by two independent observers on ten randomly selected fields observed at 63× magnification for immunofluorescence reactions by using Cell^F image analysis software (Olympus Italia, Rozzano, Italy). For immunohistochemistry, morphometric analysis was performed on 10 sections from each experimental group by means of five cases per group and using image analysis software (Olympus Italia, Rozzano Italy).

### 4.9. Statistical Analysis

The data are reported as means ± SD. Two-way Anova was used for grouped analyses and Bonferroni post-test was used to compare all groups vs. control group. The GraphPad Prism 5.0 statistical package (GraphPad Software, San Diego, CA, USA) was used for the analysis and *p <* 0.05 values were considered statistically significant.

## 5. Conclusions

These data suggest that lower expression of Dp71 is correlated with tumor progression in glioblastoma multiforme and that its expression is associated with lamin B overexpression and a high Ki-67 proliferative index.

## Figures and Tables

**Table 1:** Clinical and anatomical features of glioblastoma patients and Ki-67 tumor proliferative index.

Case	Sex	Age (Years)	Tumor Location	Ki-67	Dp71 Dystrophin Isoform (Dys) Protein Expression
1	M	54	Parieto-occipital lobe	38%	
2	M	60	Temporo-parietal lobe	34%	+
3	F	81	Left frontal lobe	45%	
4	F	42	Left temporal lobe	30%	+
5	M	58	Talamo	18%	++
6	M	68	Right parieto-occipital lobe	40%	
7	M	60	Frontal lobe	25%	++
8	F	65	Left fronto-insular	50%	
9	M	38	Temporal lobe	80%	
10	M	48	Parieto-occipital lobe	60%	
11	M	45	Left temporal lobe	25%	++
12	M	81	Left frontal lobe	28%	+
13	F	65	Right parieto-occipital lobe	15%	++
14	M	45	Frontal lobe	60%	
15	M	42	Right temporal lobe	30%	+
16	M	66	Frontal lobe	18%	++
17	F	57	Right temporal lobe	40%	
18	M	65	Frontal lobe	40%	
19	F	54	Left parieto-occipital lobe	13%	+++
20	M	73	Left parietal lobe	60%	
21	M	64	Left temporo-insular	25%	+
22	M	60	Right parietal lobe	50%	
23	F	58	Temporal lobe	30%	++
24	F	76	Left temporal lobe	15%	+++
25	M	43	Right parieto-occipital lobe	30%	+
26	M	61	Left frontal lobe	20%	++
27	F	50	Left parietal lobe	15%	++
28	M	46	Right temporo-insular	25%	+
29	M	54	Occipital lobe	80%	
30	M	74	Temporal lobe	60%	

strong (+++), moderate (++), weak (+) or absent ( ).

**Table 2:** Biorad primer PCR.

Gene	Code
*Dys*	qHsaCID0010707
*Lamin B*	qHsaC1D0014822
*Ciclophylin A*	qHsaCED0038620

Dys = Dp71 dystrophin isoform.

**Table 3:** Antibodies used for immunofluorescence (IF)/ immunohistochemistry (IH)/western blot (WB).

Protein	Catalog Number	Species	IH Diluition	IF Dilution	WB Diluition	Source
Dys	NCL-DYS2	mouse	1:5	1:10	1:100	Novacastra-Leica
Lamin B1	PA5-19468	rabbit		1:100	1:100	Thermo Fischer
Actin	Sc-47778	mouse			1:1000	SantaCruz-Bio
Alexa fluor 488 antirabbit	A11034	goat		1:300		Invitrogen
Alexa fluor 555 antimouse	A32727	goat		1:900		Invitrogen
IRDye 800CW antirabbit	926-32211	goat			1:7000	LI-COR
IRDye 800CW antimouse	926-32210	goat			1:7000	LI-COR

Dys = Dp71 dystrophin isoform.

## Abbreviations

CTRL	control
GBM	glioblastoma multiforme
DAPs	Dp71-associated proteins
Dys	Dp71 dystrophin isoform
NHA	normal human astrocytes
GLI1	glioblastoma primary cell line

## References

[1] Lederfein D., Levy Z., Augier N., Mornet D., Morris G., Fuchs O., Yaffe D., Nudel U. (1992). A 71-kilodalton protein is a major product of the Duchenne muscular dystrophy gene in brain and other nonmuscle tissues. Proc. Natl. Acad. Sci. USA.

[2] Tadayoni R., Rendon A., Soria-Jasso L.E., Cisneros B. (2012). Dystrophin dp71: The smallest but multifunctional product of the Duchenne muscular dystrophy gene. Mol. Neurobiol..

[3] Banihani R., Smile S., Yoon G., Dupuis A., Mosleh M., Snider A., McAdam L. (2015). Cognitive and neurobehavioral profile in boys with Duchenne muscular dystrophy. J. Child Neurol..

[4] Daoud F., Angeard N., Demerre B., Martie I., Benyaou R., Leturcq F., Cossee M., Deburgrave N., Saillour Y., Tuffery S. (2009). Analysis of dp71 contribution in the severity of mental retardation through comparison of Duchenne and Becker patients differing by mutation consequences on dp71 expression. Hum. Mol. Genet..

[5] Moizard M.P., Toutain A., Fournier D., Berret F., Raynaud M., Billard C., Andres C., Moraine C. (2000). Severe cognitive impairment in dmd: Obvious clinical indication for dp71 isoform point mutation screening. Eur. J. Hum. Genet..

[6] Sarig R., Mezger-Lallemand V., Gitelman I., Davis C., Fuchs O., Yaffe D., Nudel U. (1999). Targeted inactivation of dp71, the major non-muscle product of the dmd gene: Differential activity of the dp71 promoter during development. Hum. Mol. Genet..

[7] Cerna J., Cerecedo D., Ortega A., Garcia-Sierra F., Centeno F., Garrido E., Mornet D., Cisneros B. (2006). Dystrophin dp71f associates with the beta1-integrin adhesion complex to modulate pc12 cell adhesion. J. Mol. Biol..

[8] Enriquez-Aragon J.A., Cerna-Cortes J., Bermudez de Leon M., Garcia-Sierra F., Gonzalez E., Mornet D., Cisneros B. (2005). Dystrophin dp71 in pc12 cell adhesion. Neuroreport.

[9] Fort P.E., Sene A., Pannicke T., Roux M.J., Forster V., Mornet D., Nudel U., Yaffe D., Reichenbach A., Sahel J.A. (2008). Kir4.1 and aqp4 associate with dp71- and utrophin-daps complexes in specific and defined microdomains of muller retinal glial cell membrane. Glia.

[10] Villarreal-Silva M., Centeno-Cruz F., Suarez-Sanchez R., Garrido E., Cisneros B. (2011). Knockdown of dystrophin dp71 impairs pc12 cells cycle: Localization in the spindle and cytokinesis structures implies a role for dp71 in cell division. Plos ONE.

[11] Tan S., Tan J., Tan S., Zhao S., Cao X., Chen Z., Weng Q., Zhang H., Wang K., Zhou J. (2016). Decreased dp71 expression is associated with gastric adenocarcinoma prognosis. Oncotarget.

[12] Tan S., Tan S., Chen Z., Cheng K., Chen Z., Wang W., Wen Q., Zhang W. (2016). Knocking down dp71 expression in a549 cells reduces its malignancy in vivo and in vitro. Cancer Investig..

[13] Rani A.Q.M., Farea M., Maeta K., Kawaguchi T., Awano H., Nagai M., Nishio H., Matsuo M. (2019). Identification of the shortest splice variant of dp71, together with five known variants, in glioblastoma cells. Biochem. Biophys. Res. Commun..

[14] Furnari F.B., Fenton T., Bachoo R.M., Mukasa A., Stommel J.M., Stegh A., Hahn W.C., Ligon K.L., Louis D.N., Brennan C. (2007). Malignant astrocytic glioma: Genetics, biology, and paths to treatment. Genes Dev..

[15] Pearson J.R.D., Regad T. (2017). Targeting cellular pathways in glioblastoma multiforme. Signal Transduct. Target. Ther..

[16] Wen P.Y., Kesari S. (2008). Malignant gliomas in adults. New Engl. J. Med..

[17] Villarreal-Silva M., Suarez-Sanchez R., Rodriguez-Munoz R., Mornet D., Cisneros B. (2010). Dystrophin dp71 is critical for stability of the daps in the nucleus of pc12 cells. Neurochem. Res..

[18] Nico B., Paola Nicchia G., Frigeri A., Corsi P., Mangieri D., Ribatti D., Svelto M., Roncali L. (2004). Altered blood-brain barrier development in dystrophic mdx mice. Neuroscience.

[19] Noell S., Wolburg-Buchholz K., Mack A.F., Ritz R., Tatagiba M., Beschorner R., Wolburg H., Fallier-Becker P. (2012). Dynamics of expression patterns of aqp4, dystroglycan, agrin and matrix metalloproteinases in human glioblastoma. Cell Tissue Res..

[20] Nico B., Ribatti D. (2011). Role of aquaporins in cell migration and edema formation in human brain tumors. Exp. Cell Res..

[21] Nico B., Mangieri D., Tamma R., Longo V., Annese T., Crivellato E., Pollo B., Maderna E., Ribatti D., Salmaggi A. (2009). Aquaporin-4 contributes to the resolution of peritumoural brain oedema in human glioblastoma multiforme after combined chemotherapy and radiotherapy. Eur. J. Cancer.

[22] Calogero A., Pavoni E., Gramaglia T., D’Amati G., Ragona G., Brancaccio A., Petrucci T.C. (2006). Altered expression of alpha-dystroglycan subunit in human gliomas. Cancer Biol. Ther..

[23] Marquez F.G., Cisneros B., Garcia F., Ceja V., Velazquez F., Depardon F., Cervantes L., Rendon A., Mornet D., Rosas-vargas H. (2003). Differential expression and subcellular distribution of dystrophin dp71 isoforms during differentiation process. Neuroscience.

[24] Acosta R., Montanez C., Fuentes-Mera L., Gonzalez E., Gomez P., Quintero-Mora L., Mornet D., Alvarez-Salas L.M., Cisneros B. (2004). Dystrophin dp71 is required for neurite outgrowth in pc12 cells. Exp. Cell Res..

[25] Suarez-Sanchez R., Aguilar A., Wagstaff K.M., Velez G., Azuara-Medina P.M., Gomez P., Vasquez-Limeta A., Hernandez-Hernandez O., Lieu K.G., Jans D.A. (2014). Nucleocytoplasmic shuttling of the duchenne muscular dystrophy gene product dystrophin dp71d is dependent on the importin alpha/beta and crm1 nuclear transporters and microtubule motor dynein. Biochim. Et Biophys. Acta.

[26] Dauer W.T., Worman H.J. (2009). The nuclear envelope as a signaling node in development and disease. Dev. Cell.

[27] Dauer W.T., Worman H.J. (2010). New messages in the nuclear envelope. Cell Cycle.

[28] Dechat T., Adam S.A., Taimen P., Shimi T., Goldman R.D. (2010). Nuclear lamins. Cold Spring Harb. Perspect. Biol..

[29] Dittmer T.A., Misteli T. (2011). The lamin protein family. Genome Biol..

[30] Butin-Israeli V., Adam S.A., Goldman A.E., Goldman R.D. (2012). Nuclear lamin functions and disease. Trends Genet..

[31] Irianto J., Pfeifer C.R., Ivanovska I.L., Swift J., Discher D.E. (2016). Nuclear lamins in cancer. Cell. Mol. Bioeng..

[32] Sakthivel K.M., Sehgal P. (2016). A novel role of lamins from genetic disease to cancer biomarkers. Oncol. Rev..

[33] Broers J.L., Raymond Y., Rot M.K., Kuijpers H., Wagenaar S.S., Ramaekers F.C. (1993). Nuclear a-type lamins are differentially expressed in human lung cancer subtypes. Am. J. Pathol..

[34] Moss S.F., Krivosheyev V., de Souza A., Chin K., Gaetz H.P., Chaudhary N., Worman H.J., Holt P.R. (1999). Decreased and aberrant nuclear lamin expression in gastrointestinal tract neoplasms. Gut.

[35] Coradeghini R., Barboro P., Rubagotti A., Boccardo F., Parodi S., Carmignani G., D’Arrigo C., Patrone E., Balbi C. (2006). Differential expression of nuclear lamins in normal and cancerous prostate tissues. Oncol. Rep..

[36] Li L., Du Y., Kong X., Li Z., Jia Z., Cui J., Gao J., Wang G., Xie K. (2013). Lamin b1 is a novel therapeutic target of betulinic acid in pancreatic cancer. Clin. Cancer Res..

[37] Lim S.O., Park S.J., Kim W., Park S.G., Kim H.J., Kim Y.I., Sohn T.S., Noh J.H., Jung G. (2002). Proteome analysis of hepatocellular carcinoma. Biochem. Biophys. Res. Commun..

[38] Sun S., Xu M.Z., Poon R.T., Day P.J., Luk J.M. (2010). Circulating lamin b1 (lmnb1) biomarker detects early stages of liver cancer in patients. J. Proteome Res..

[39] Louis D.N., Ohgaki H., Wiestler O.D., Cavenee W.K., Burger P.C., Jouvet A., Scheithauer B.W., Kleihues P. (2007). The 2007 who classification of tumours of the central nervous system. Acta Neuropathol..

